# Supplementary material for: Identification of immune-related mechanisms of cetuximab induced skin toxicity in colorectal cancer patients
Source: PLoS One. 2022 Oct 21;17(10):e0276497. doi: 10.1371/journal.pone.0276497 (PMC9586384; doi:10.1371/journal.pone.0276497)
Supplement: S1 Raw data — (ZIP) [file pone.0276497.s002.zip › RFLP_total data.pptx]

## Slide 1
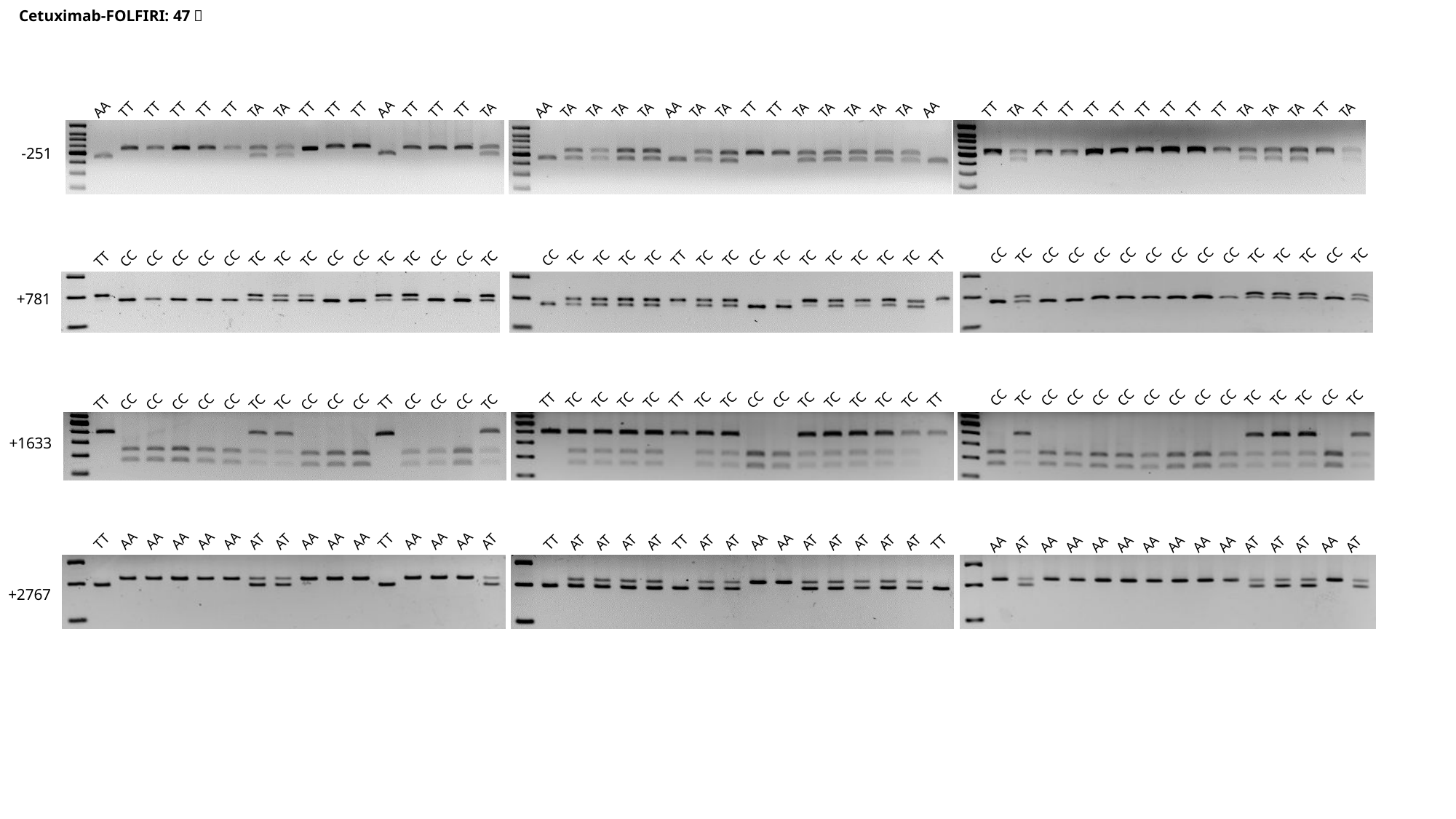

Cetuximab-FOLFIRI: 47명
AA
TT
TT
TT
TT
TT
TA
TA
TT
TT
TT
AA
TT
TT
TT
TA
AA
TA
TA
TA
TA
AA
TA
TA
TT
TT
TA
TA
TA
TA
TA
AA
TT
TA
TT
TT
TT
TT
TT
TT
TT
TT
TA
TA
TA
TT
TA
-251
TC
CC
CC
CC
CC
CC
CC
CC
CC
CC
TC
TC
TC
CC
TC
TC
CC
TC
TC
TC
TT
TC
TC
CC
TC
TC
TC
TC
TC
TC
TT
TT
CC
CC
CC
CC
CC
TC
TC
TC
CC
CC
TC
TC
CC
CC
TC
+781
CC
TC
CC
CC
CC
CC
CC
CC
CC
CC
TC
TC
TC
CC
TC
TT
TC
TC
TC
TC
TT
TC
TC
CC
CC
TC
TC
TC
TC
TC
TT
TT
CC
CC
CC
CC
CC
TC
TC
CC
CC
CC
TT
CC
CC
CC
TC
+1633
TT
AA
AA
AA
AA
AA
AT
AT
AA
AA
AA
TT
AA
AA
AA
AT
TT
AT
AT
AT
AT
TT
AT
AT
AA
AA
AT
AT
AT
AT
AT
TT
AA
AT
AA
AA
AA
AA
AA
AA
AA
AA
AT
AT
AT
AA
AT
+2767

## Slide 2
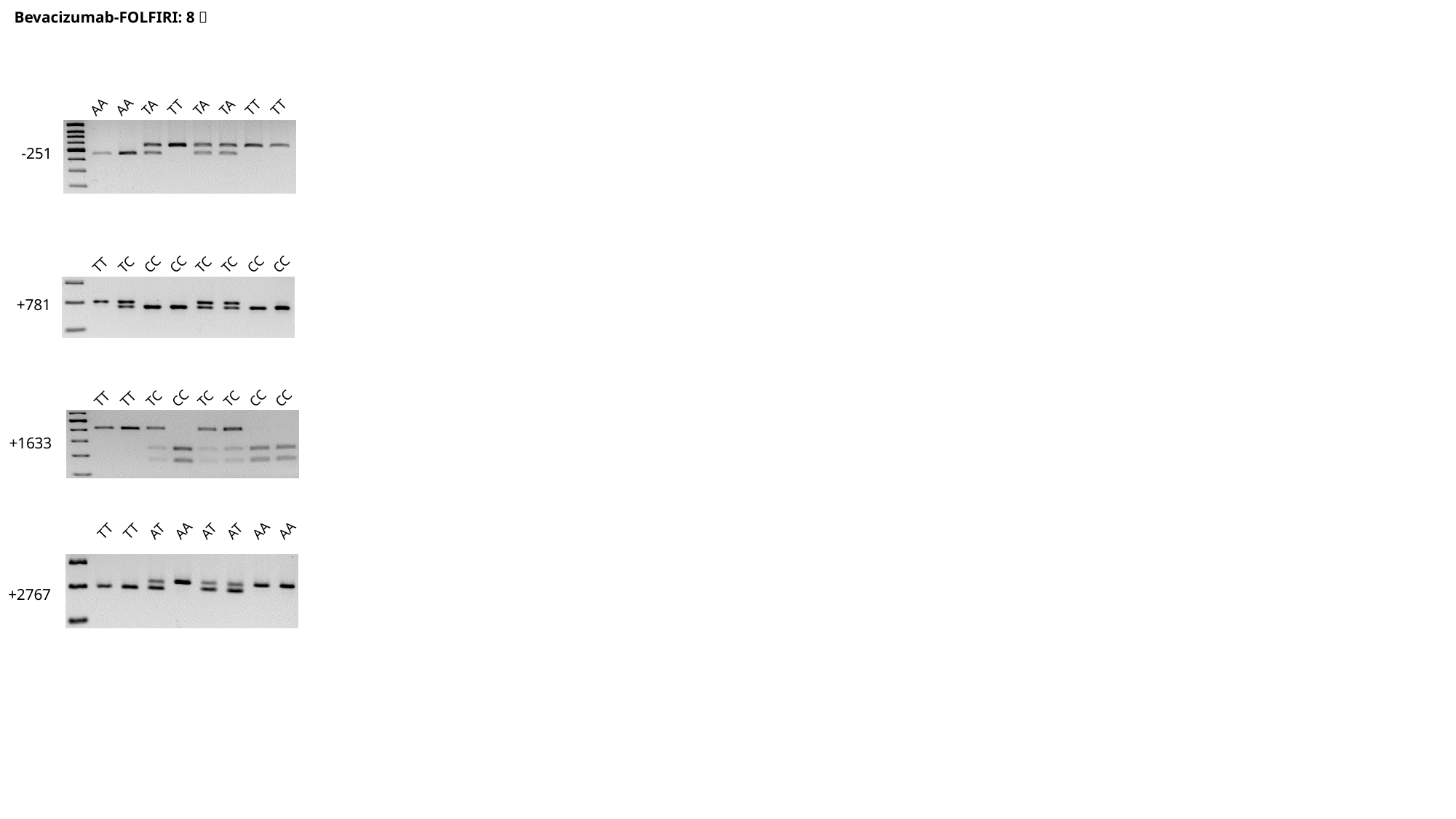

Bevacizumab-FOLFIRI: 8명
AA
AA
TA
TT
TA
TA
TT
TT
-251
TT
TC
CC
CC
TC
TC
CC
CC
+781
TT
TT
TC
CC
TC
TC
CC
CC
+1633
TT
TT
AT
AA
AT
AT
AA
AA
+2767
