# Supplementary material for: Identification of immune-related mechanisms of cetuximab induced skin toxicity in colorectal cancer patients
Source: PLoS One. 2022 Oct 21;17(10):e0276497. doi: 10.1371/journal.pone.0276497 (PMC9586384; doi:10.1371/journal.pone.0276497)
Supplement: Attachment — Submitted filename: Response.docx [file pone.0276497.s003.docx]

Authors’ reply

Journal Requirements:

1. Please ensure that your manuscript meets PLOS ONE's style requirements, including those for file naming.

Answer: We have reviewed the style requirement, and updated the manuscript and files.

2. In your Data Availability statement, you have not specified where the minimal data set underlying the results described in your manuscript can be found. PLOS defines a study's minimal data set as the underlying data used to reach the conclusions drawn in the manuscript and any additional data required to replicate the reported study findings in their entirety.

Answer: We will upload the original images and dataset files with the revision

3. We note that you have stated that you will provide repository information for your data at acceptance. Should your manuscript be accepted for publication, we will hold it until you provide the relevant accession numbers or DOIs necessary to access your data. If you wish to make changes to your Data Availability statement, please describe these changes in your cover letter and we will update your Data Availability statement to reflect the information you provide.

Answer: We will upload the original images and dataset files with the revision. We do not have other repository to upload the data.

4. PLOS ONE now requires that authors provide the original uncropped and unadjusted images underlying all blot or gel results reported in a submission’s figures or Supporting Information files.

Answer: We will upload the original images and dataset files with the revision.

5. PLOS requires an ORCID iD for the corresponding author in Editorial Manager on papers submitted after December 6th, 2016. Please ensure that you have an ORCID iD and that it is validated in Editorial Manager.

Answer: We have updated the ORCID ID for the corresponding author.

Reviewers' comments:

Reviewer #1: Authors performed an interesting analysis regarding a potential mechanism at the basis of anti-EGFR related skin toxicity. The paper is well written although some english typo have to be assessed.

Considering the small sample size of patients it would be interesting describe if patients with high grade skin toxicity have experienced a better survival compared with patients with no or low grade skin toxicity that received the same treatment (folfiri cetuximab).

Moreover, besides IL-8, have some other changes in the other 19 cytokines been identyfied?

Answer: Thank you very much for your comments. We had an English editing before we submitted the manuscript last year. As you mentioned, the number of the patients in this study was small (N = 38), and only 30 patients were exposed to cetuximab based chemotherapy. When we compared the survival according to the skin rash, there was no significant difference. We believe that we do not have statistical power due to small number of the patients.

Thank you very much for your kind comment.

End of document.
