# Supplementary material for: Identification of immune-related mechanisms of cetuximab induced skin toxicity in colorectal cancer patients
Source: PLoS One. 2022 Oct 21;17(10):e0276497. doi: 10.1371/journal.pone.0276497 (PMC9586384; doi:10.1371/journal.pone.0276497)
Supplement: Attachment — Submitted filename: Response2.docx [file pone.0276497.s004.docx]

Authors’ reply

Additional Editor Comments (if provided):

The reviewer opines that the comments/suggestions were not rebutted completely. Kindly make a point-wise response to the reviewer's comments.

Answer: We are providing the response to the reviewer’s comment in the following paragraph.

[Note: HTML markup is below. Please do not edit.]

Reviewers' comments:

Reviewer's Responses to Questions

Comments to the Author

1. If the authors have adequately addressed your comments raised in a previous round of review and you feel that this manuscript is now acceptable for publication, you may indicate that here to bypass the “Comments to the Author” section, enter your conflict of interest statement in the “Confidential to Editor” section, and submit your "Accept" recommendation.

Reviewer #1: (No Response)

Answer: Not applicable

2. Is the manuscript technically sound, and do the data support the conclusions?

The manuscript must describe a technically sound piece of scientific research with data that supports the conclusions. Experiments must have been conducted rigorously, with appropriate controls, replication, and sample sizes. The conclusions must be drawn appropriately based on the data presented.

Reviewer #1: (No Response)

Answer: Not applicable

3. Has the statistical analysis been performed appropriately and rigorously?

Reviewer #1: (No Response)

Answer: Not applicable

4. Have the authors made all data underlying the findings in their manuscript fully available?

The PLOS Data policy requires authors to make all data underlying the findings described in their manuscript fully available without restriction, with rare exception (please refer to the Data Availability Statement in the manuscript PDF file). The data should be provided as part of the manuscript or its supporting information, or deposited to a public repository. For example, in addition to summary statistics, the data points behind means, medians and variance measures should be available. If there are restrictions on publicly sharing data—e.g. participant privacy or use of data from a third party—those must be specified.

Reviewer #1: (No Response)

Answer: Not applicable

5. Is the manuscript presented in an intelligible fashion and written in standard English?

PLOS ONE does not copyedit accepted manuscripts, so the language in submitted articles must be clear, correct, and unambiguous. Any typographical or grammatical errors should be corrected at revision, so please note any specific errors here.

Reviewer #1: (No Response)

Answer: Not applicable

6. Review Comments to the Author

Please use the space provided to explain your answers to the questions above. You may also include additional comments for the author, including concerns about dual publication, research ethics, or publication ethics. (Please upload your review as an attachment if it exceeds 20,000 characters)

Reviewer #1: Authors have not answered to a point by point review.

Could they please give comments regarding my specific questions? even if the number of patients is low I would like to see what I have requested.

Otherwise I can not accept the paper for publication

Answer: As the reviewer suggested, now we added the survival analyses according the presence or grades of skin rash in the manuscript (page 9, line number 166 – 172). We are also providing the Kaplan-Meier survival curves to respond the reviewer’s comments properly.

Figure 1. Overall survival for all patients.

Figure 2. Overall survival according to the skin rash (1=patients with skin rash, 2=patients without skin rash)

| skin_rash | Median | | | | | | | |
| --- | --- | --- | --- | --- | --- | --- | --- | --- |
|  | Estimate | Std. Error | | 95% Confidence Interval | | | | |
|  |  |  |  | Lower Bound | | Upper Bound | | |
| 1.00 | 1064.000 | 331.513 | | 414.234 | | 1713.766 | | |
| 2.00 | 888.000 | 57.991 | | 774.338 | | 1001.662 | | |
| Overall | 1064.000 | 319.232 | | 438.304 | | 1689.696 | | |
| **Overall Comparisons** | | | | | | | |  |
|  | | | Chi-Square | | df | | Sig. |  |
| Log Rank (Mantel-Cox) | | | .041 | | 1 | | .840 |  |
| Test of equality of survival distributions for the different levels of skin_rash. | | | | | | | |  |

Figure 3. Overall survival according to the grade of skin rash

| skin_rash_grade | Median | | | | | | | |
| --- | --- | --- | --- | --- | --- | --- | --- | --- |
|  | Estimate | | Std. Error | | 95% Confidence Interval | | | |
|  |  |  |  |  | Lower Bound | | Upper Bound | |
| 1.00 | 807.000 | | 69.296 | | 671.179 | | 942.821 | |
| 2.00 | 1275.000 | | 216.375 | | 850.906 | | 1699.094 | |
| 3.00 | 1729.000 | | 593.444 | | 565.851 | | 2892.149 | |
| Overall | 1064.000 | | 331.513 | | 414.234 | | 1713.766 | |
| **Overall Comparisons** | | | | | | | |  |
|  | | Chi-Square | | df | | Sig. | |  |
| Log Rank (Mantel-Cox) | | 1.277 | | 2 | | .528 | |  |
| Test of equality of survival distributions for the different levels of skin_rash_grade. | | | | | | | |  |

Another comment from the reviewer was as follows:

“Moreover, besides IL-8, have some other changes in the other 19 cytokines been identyfied?”

Answer: In the table 3, we already showed the changes of 20 cytokines levels in detail. Besides IL-8, we highlighted the changes with statistical significance in the manuscript (page 11, line number 181 – 175). the mean serum IL-1 alpha and MIP-1 beta levels for patients in group 3 (bevacizumab treated) was lower, but there was no difference between patients with skin rash and those without skin rash.

7. PLOS authors have the option to publish the peer review history of their article (what does this mean?). If published, this will include your full peer review and any attached files.

If you choose “no”, your identity will remain anonymous but your review may still be made public.

Do you want your identity to be public for this peer review? For information about this choice, including consent withdrawal, please see our Privacy Policy.

Reviewer #1: NoThank you very much for your kind comment.

Answer: We respect the reviewer’s decision

End of document.
